# Transcriptomic Analysis Reveals the Mechanisms of Cadmium Transport and Detoxification in Portuguese Oysters (*Crassostrea angulata*)

**DOI:** 10.3390/ani15071041

**Published:** 2025-04-03

**Authors:** Kairui Qin, Longping Wu, Shixing Fu, Huayong Que, Bo Shi

**Affiliations:** State Key Laboratory of Mariculture Breeding, Fisheries College, Jimei University, Xiamen 361005, China; 202211908044@jmu.edu.cn (K.Q.); longpingwu@163.com (L.W.); 202211908040@jmu.edu.cn (S.F.)

**Keywords:** Portuguese oysters, Cd accumulation, individual variation, transcriptome, regulatory genes

## Abstract

Oysters are a globally distributed aquatic economic shellfish with a remarkable ability to accumulate cadmium (Cd). The phenomenon of Cd content exceeding safety standards in oysters occurs frequently, posing a significant risk to food safety. In this study, Portuguese oysters (*Crassostrea angulata*) were exposed to 2 μg/L of Cd for 15 days. Individuals with significantly different Cd accumulation were selected for transcriptomic sequencing analysis. KEGG enrichment analysis revealed that the differentially expressed genes (DEGs) between high- and low-Cd-accumulation individuals, as well as those before and after Cd exposure, were primarily enriched in metabolic pathways (33.8%) and organismal systems (32.2%). ABC transporters, phagosomes, glutathione metabolism, and the biosynthesis of amino acids played crucial roles in Cd accumulation and detoxification processes. Metal cation transport-related genes, including *zip1*, *copt5.1*, and *orct2*, may be involved in the Cd transport process in Portuguese oysters, and their differential expression influences Cd accumulation in the soft tissues. Meanwhile, genes such as *sod3*, *cyp4f22*, and *abca3* are likely to play significant roles in detoxification under Cd exposure. Additionally, alternative splicing analysis identified 13 potential genes associated with Cd response in Portuguese oysters, including *cs2*, *gfpt1*, and *acox1*.

## Simple Summary

The occurrence of excessive cadmium (Cd) levels in oysters is a recurrent issue, posing a critical threat to food safety. In the present study, through a comprehensive transcriptome analysis, we delved into the disparities in Cd transport and detoxification mechanisms between high- and low-Cd-accumulating individuals. Our findings revealed that the differential expression of divalent metal ion transporters, such as *zip1*, *orct2*, and *copt5.1*, might be a pivotal determinant contributing to the remarkable variations in Cd enrichment among individual Portuguese oysters (*Crassostrea angulata*). Concurrently, genes including *sod3*, *cyp4f22*, and *abca3* were found to play indispensable roles in the Cd detoxification pathway. The identification of these key regulatory genes involved in Cd transport and detoxification not only furnishes a solid theoretical underpinning for the subsequent breeding of novel Portuguese oyster varieties with low Cd accumulation but also represents a crucial stride towards safeguarding food safety.

## 1. Introduction

With the rapid development of global industry and agriculture, large amounts of heavy metals such as copper (Cu), zinc (Zn), cadmium (Cd), and lead (Pb) have been discharged into the ocean, causing severe adverse effects on the stability of marine ecosystems and the survival and distribution of aquatic organisms [1,2,3]. Cd is a typical non-essential heavy metal pollutant for organisms that is widely distributed in the atmosphere, soil, and oceans. It is characterized by its high accumulation potential, long half-life, and high mobility [4,5,6]. Studies have shown that Cd can cause serious oxidative damage to organisms, disrupt the balance of divalent metal ions within cells, and lead to metabolic disorders and protein dysfunction [7,8,9]. Furthermore, Cd can accumulate in higher trophic-level organisms through the food chain. High concentrations of Cd accumulation can damage the structure and function of organs such as the liver, kidneys, and testes, thereby inhibiting growth and the development of reproductive cells [10,11,12]. Additionally, the International Agency for Research on Cancer (IARC) of the World Health Organization classified Cd and Cd compounds as Group 1 human carcinogens in 2017. Several human cancers, such as breast cancer, kidney cancer, lung cancer, and pancreatic cancer, have been linked to Cd exposure [13,14,15].

Due to their filter-feeding and sessile lifestyle, combined with an extremely imbalanced cadmium (Cd) intake/elimination ratio, oysters can accumulate Cd concentrations exceeding 1000-fold that of ambient water, with a bioconcentration factor (BCF) reaching up to 47,946 [16,17,18,19,20]. Despite such high Cd accumulation, oyster tissues exhibit no obvious malformations or pathological alterations, as they employ translocation, sequestration, and compartmentalization mechanisms to mitigate Cd toxicity [21]. Studies have confirmed that 67.6–87.8% of Cd in oyster tissues is bioavailable to humans. The prolonged consumption of Cd-contaminated oysters significantly elevates blood Cd levels in humans, thereby increasing the risk of cancer [22,23,24,25,26]. Due to the occurrence of Cd concentrations exceeding regulatory standards in cultured oysters, reducing Cd accumulation capacity is a critical objective for improving aquaculture quality and ensuring food safety. Notably, substantial individual variation in Cd accumulation exists among oysters, with Cd concentrations differing by 5- to 7-fold within populations under identical environmental conditions. Genetic studies indicate that Cd accumulation in oysters is a quantitative trait with a broad-sense heritability of 0.53 ± 0.11 [27]. Therefore, investigations into the molecular mechanisms underlying Cd accumulation and tolerance in oysters, as well as the identification of key regulatory genes, will provide a theoretical foundation for the genetic improvement of Cd accumulation traits in aquaculture.

As a non-essential element, Cd can enter cells through other metal transport channels, while organisms employ mechanisms such as cell wall modification, reactive oxygen species (ROS) detoxification, vacuolar compartmentalization, and efflux to mitigate its toxicity [20,28]. The current understanding of the molecular mechanisms underlying Cd accumulation and tolerance in bivalves and other aquatic animals remains limited, with most studies focusing solely on gene/protein expression changes before and after Cd exposure. For instance, Hu et al. [29] demonstrated that the upregulated expression of *sod1*, *cat*, and *gpx* enhanced ROS detoxification in zebrafish (*Danio rerio*) under Cd stress. Dong et al.’s study [30] reported that high expression of ABC transporters (*abca1*, *abcb1*, *abcc3*), glutathione metabolism-related genes (*pepn*, *lap3*, *mgst3*), and energy metabolism pathways (*dodecanoic acid*, *acetyl-coa*, *acp*) improved Cd tolerance in the Pacific Oyster (*Crassostrea gigas*). Additionally, Zhao et al. [31] used gene knockout technology to show that *c-myc* knockout not only reduced stress and detoxification gene expression in blood clams (*Tegillarca granosa*) during Cd exposure but also suppressed igf1r expression, inhibiting cell proliferation. Most existing studies focus on acute response mechanisms at different Cd exposure concentrations or time points, whereas comparative analyses of individuals with divergent Cd accumulation capacities remain scarce. In fact, comparative studies of individuals with significantly differentiated traits are critical for identifying key genes and pathways regulating Cd accumulation. For example, transcriptomic analyses of Ostrea rivularis (*Crassostrea ariakensis*) populations with distinct glycogen content revealed that differentially expressed genes (DEGs) were enriched in starch and sucrose metabolism and the insulin signaling pathway. The high expression of *agl*, *pygm*, and *gys2* likely promotes glycogen accumulation by enhancing glycogen anabolic pathways [32]. Similarly, studies on young largemouth bronze gudgeon (*Coreius guichenoti*) with divergent hepatic lipid content showed that the upregulation of lipid synthesis genes and the downregulation of lipid degradation genes may disrupt normal lipid metabolism, leading to hepatic steatosis through increased fat deposition [33].

Portuguese oysters, also known as Fujian oysters, are a globally distributed economic shellfish species. In China, they are mainly found in the southern coastal regions, such as Fujian and Zhejiang provinces. Its aquaculture production accounts for approximately 50% of the total oyster aquaculture production in China [34]. In our previous research, we conducted a genome-wide association study (GWAS) on Cd accumulation traits in Portuguese oysters [35]. From 141 SNPs significantly associated with Cd accumulation traits, genes such as *zip1*, *zip3*, *slc46a1*, and *slc46a3* were identified, suggesting their potential involvement in the regulation of Cd accumulation in Portuguese oysters. Building on this foundation, this study conducts a comparative transcriptomic analysis of Portuguese oysters with significantly different Cd accumulation capacities (high-Cd-accumulating and low-Cd-accumulating individuals) to explore the mechanisms underlying these differences and identify key regulatory genes involved in Cd transport and detoxification. This research aims to provide a theoretical basis for the breeding of new Portuguese oyster varieties with low Cd accumulation.

## 2. Materials and Methods

### 2.1. Laboratory Cd Exposure

The Portuguese oysters used in the experiment were one-year-old individuals from the same batch cultured in Lianjiang, Fujian Province. After 1000 oysters were brought to the laboratory, surface attachments were removed, and the oysters were evenly distributed into two aquaculture tanks (3000 L) for a 7-day acclimation period. During the temporary cultivation period, sufficient dissolved oxygen was maintained. The water was completely replaced once a day. Dead individuals were promptly removed. Chlorella (self-cultured) was fed at a rate of 2% of the dry weight of the oyster soft tissues. The salinity (29 ± 0.5‰) and temperature (26 ± 0.5 °C) of the seawater were measured using an electronic salinometer AZ8371 (Shenzhen Graigar Technology Co., Ltd., Shenzhen, China). For the preparation of 10 ppm Cd ion stock solution (CdCl_2_; molecular weight: 183.32 g/mol, Sigma Aldrich, St. Louis, MO, USA), dissolve an appropriate amount of CdCl_2_ in Milli-Q water. After acclimation, 500 oysters with similar sizes (shell length: 72.4–82.5 mm) and healthy status were selected from culture tanks for a 15-day aqueous Cd exposure experiment in three 500 L tanks at 2 μg/L. Management protocols during the experiment were consistent with the acclimation period. Daily full water exchange was followed by adjusting seawater Cd concentration using a pre-prepared Cd ion stock solution. On Days 0 and 15 of exposure, 50 oysters per tank were randomly sampled, dissected to obtain mantle and visceral mass tissues, and rinsed with physiological saline. Each tissue sample was divided into two aliquots: one was oven-dried for Cd content analysis, and the other was flash-frozen in liquid nitrogen for storage at −80 °C prior to transcriptome sequencing.

### 2.2. Cd Content Determination and Sample Selection

Based on the method described by Maanan et.al. [36] for determining metal content in oysters, the Cd concentrations in the mantle and visceral mass of 50 selected Portuguese oyster individuals were measured on Day 0 and Day 15 using an Agilent ICP-MS-7700 (Agilent, Santa Clara, CA, USA). The specific procedure was as follows: The mantle and visceral mass of each oyster were dried to a constant weight at 80 °C and weighed. The dried tissues (approximately 0.1 g) were then digested with concentrated nitric acid (analytical grade) at room temperature for 12 h and then heated at 80 °C for another 12 h until complete digestion. After appropriate dilution, the Cd content was measured using ICP-MS. A multi-element standard (Agilent) was used for external calibration, with germanium (Ge) as the internal standard to correct for instrument drift and sensitivity changes. Every 20 samples were repeated for quality control, and the normality of Cd content was tested using SPSS 24 software. Finally, three individuals with the lowest Cd concentrations in both mantle and visceral mass at each time point were defined as low-Cd-accumulating individuals (LC), while three with the highest Cd concentrations in both tissues were classified as high-Cd-accumulating individuals (HC).

### 2.3. RNA Extraction and Transcriptome Sequencing

The mantles and visceral masses from three LC and three HC specimens, harvested on Day 0 and Day 15, respectively, were selected for subsequent transcriptome sequencing analysis (total 24). Total RNA was extracted from each sample using TRIzol^®^ reagent (Magen, Guangzhou, China). The concentration, purity, and integrity of the RNA were evaluated using an Agilent 2100 Bioanalyzer (Agilent, Santa Clara, CA, USA) and agarose gel electrophoresis. Qualified samples that passed the quality inspection were used to construct libraries with the ABclonal mRNA-seq Lib Prep Kit (ABclonal, Wuhan, China) as follows: The mRNA was purified from 1 μg total RNA using oligo (dT) magnetic beads, followed by fragmentation carried out using divalent cations at elevated temperatures in ABclonal First Strand Synthesis Reaction Buffer. Subsequently, first-strand cDNAs were synthesized with random hexamer primers and Reverse Transcriptase (RNase H) using mRNA fragments as templates, followed by second-strand cDNA synthesis using DNA polymerase I, RNAse H, buffer, and dNTPs. The synthesized double-stranded cDNA fragments were then adapter-ligated for preparation of the paired-end library. Adaptor-ligated cDNA was used for PCR amplification. PCR products were purified (AMPure XP system), and library quality was assessed on an Agilent Bioanalyzer 4150 system (Agilent Technologies Inc., Palo Alto, CA, USA). Finally, sequencing was performed using an Illumina NovaSeq 6000/MGISEQ-T7 instrument (Illumina/BGI, San Diego, CA, USA). RNA extraction, library preparation, and sequencing were all entrusted to the company Sinotech New Life Biotechnology Co., Ltd. (Shanghai, China).

After quality control processing of the raw sequencing data, the HISAT2 software (http://daehwankimlab.github.io/hisat2/ (accessed on 4 March 2022)) was used to map the clean reads to the reference genome to obtain mapped reads. The FeatureCounts software (http://subread.sourceforge.net/ (accessed on 4 March 2022)) was used to count the number of reads mapped to each gene. Then, based on the length of the gene and the read count mapped to the gene, the fragments per kilobase of exon per million fragments mapped (FPKM) for each gene were calculated. According to different objectives, we classified comparison groups into two categories: (1) to identify genes regulating Cd accumulation, HC was used as the control group and LC as the experimental group in same-day comparison groups; and (2) to characterize Cd-responsive genes (transporters and detoxification), HC was used as the control and LC as the experimental group in the pre- and post-Cd-exposure comparison groups. Differential expression analysis was performed using the DESeq2 software (http://bioconductor.org/packages/release/bioc/html/DESeq2.html (accessed on 4 March 2022)) with a fold change threshold of |log2FC| > 1 and an adjusted *p*-value < 0.05. Then, a volcano plot was drawn to visualize the overall distribution of DEGs. KEGG enrichment analysis was carried out to identify important pathways or key genes involved in the process of Portuguese oysters’ adaptation to Cd exposure, which was performed using the ClusterProfiler R package 4.6.0 (*p*-value < 0.05). According to the degree of Cd accumulation, each comparison group was classified into a low-Cd-accumulating group and a high-Cd-accumulating group (Table 1).

### 2.4. Screening of Cd Transport and Detoxification-Related Genes and GWAS Joint Analysis

Based on our previous GWAS result [37], we compared 261 genes that were significantly associated with Cd accumulation traits with the DEGs from the transcriptome analysis to identify co-localized genes. Potential functional genes related to Cd transport and detoxification in Portuguese oysters were screened based on previous studies, KEGG enrichment analysis, and gene expression levels in the transcriptome data.

### 2.5. Alternative Splicing Analysis

Alternative splicing (AS) is an important mechanism for regulating gene expression and protein diversity, which contributes to the significant differences in gene and protein numbers in eukaryotes. This study used rMATS software (http://rnaseq-mats.sourceforge.net/index.html (accessed on 4 March 2022)) for statistical, quantitative, and differential analysis. rMATS classifies AS events into five categories: skipped exon (SE), mutually exclusive exons (MXE), alternative 5′ splice site (A5SS), alternative 3′splice site (A3SS), and retained intron (RI). The KEGG enrichment analysis of genes with differential AS events was performed using the ClusterProfiler R package.

### 2.6. Real-Time Quantitative PCR Analysis

Twelve potential key genes related to Cd accumulation and detoxification in Portuguese oysters were selected from the transcriptome analysis results for validation using real-time quantitative PCR (*qPCR*). The Elongation Factor 1α gene (*ef1-α*) [38] of Portuguese oysters was used as a reference gene. According to the primer design principles for *qPCR*, primers for each candidate gene were designed using the NCBI Primer-BLAST tool (Table 2). The amplification length was controlled within 100–250 bp. The synthesis of primers was completed by Sangon Biotech (Shanghai) Co. (Shanghai, China), Ltd. The HiScript III RT SuperMix for *qPCR* (+gDNA wiper) kit (Vazyme, Nanjing, China) was used to reverse-transcribe the RNA of each sample. The 2^−ΔΔCt^ method was adopted to calculate the relative expression levels of genes. The reaction system (20 μL) contained Taq Pro Universal SYBR *qPCR* Master Mix (Vazyme, Nanjing, China) (10 μL), forward and reverse primers (0.4 μL each), DEPC-treated water (7.2 μL), and cDNA (2 μL). The *qPCR* reaction program was as follows: 95 °C for 30 s (pre-denaturation), followed by 40 cycles of 95 °C for 10 s and 60 °C for 30 s (cyclic reaction), with a final step of 95 °C for 15 s, 60 °C for 60 s, and 95 °C for 15 s (dissociation curve).

## 3. Results

### 3.1. Cd Content Determination

The Cd content determination results (Table 3) showed that on Day 0 of Cd exposure, the Cd content in the mantle and visceral mass of Portuguese oysters ranged from 2.92 to 7.97 µg/g dry weight (DW) and 5.71 to 21.87 µg/g DW, respectively. After 15 days of Cd exposure, the Cd content in the mantle and visceral mass increased to 8.65–58.84 µg/g DW and 13.00–43.92 µg/g DW, respectively, representing 4.49-fold and 1.81-fold increases compared to the initial levels. The corresponding bioaccumulation factors (BAFs) were quantified as 8.17 and 5.23 for the mantle and visceral mass, respectively. These results indicate that both tissues can significantly accumulate Cd, with the mantle exhibiting a stronger accumulation capacity than the visceral mass. The Cd content in LC and HC individuals is shown in Table 4. On Day 0, the Cd content in the mantle and visceral mass of LC individuals was 3.48 µg/g DW and 7.13 µg/g DW, respectively, while in HC individuals, it was 7.32 µg/g DW and 21.22 µg/g DW, respectively (2.1-fold and 2.98-fold higher than LC). After 15 days of Cd exposure, the Cd content in the mantle and visceral mass of LC individuals reached 9.54 µg/g DW and 13.49 µg/g DW, respectively, whereas in HC individuals, it increased to 48.16 µg/g DW and 34.29 µg/g DW, respectively (5.05-fold and 2.54-fold higher than LC).

### 3.2. Sequencing Data Quality

Transcriptome sequencing results for 24 samples (Supplementary Materials) showed that the raw reads ranged from 39,332,878 to 80,189,660. After quality control, we obtained a total of 179.31 G of high-quality clean data from 24 samples. The minimum value of Q30 was 91.84%, and the GC content ranged from 42.47% to 47.65%. The clean reads were mapped to the Portuguese oyster reference genome, with a total mapped rate of over 80% for all libraries (80.42–85%). These results indicate that the sequencing data quality was high and suitable for subsequent transcriptomic analysis.

### 3.3. Number of Differentially Expressed Genes

**Figure 1:**
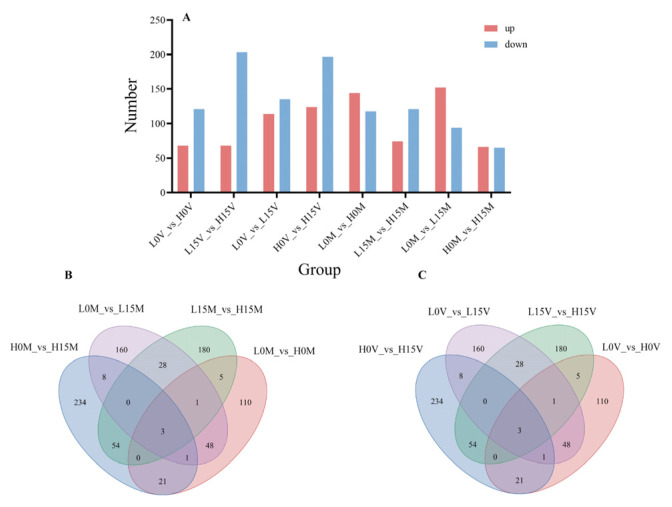
Differentially expressed genes. (**A**) Up-(red) and down-regulated (blue) DEGs in each comparison group. (**B**) Venn diagram of DEGs in four comparison groups in the mantle. (**C**) Venn diagram of DEGs in four comparison groups in the visceral mass.

The differential expression analysis results for each comparison group are shown in Figure 1. In the mantle of Portuguese oysters, 262 and 195 DEGs were identified in the LC and HC groups on Day 0 (L0M_vs_H0M) and Day 15 (L15M_vs_H15M) of Cd exposure, respectively, with 144 and 74 upregulated DEGs and 118 and 121 downregulated DEGs, respectively. There were eight common DEGs between the two groups. In the comparison groups between Day 0 and Day 15, the low-Cd-accumulating comparison group (L0M_vs_L15M) and the high-Cd-accumulating comparison group (H0M_vs_H15M) identified 246 and 131 DEGs, respectively, with 152 and 66 upregulated DEGs and 94 and 65 downregulated DEGs, respectively. There were five common DEGs between the two groups. In the visceral mass, 189 and 271 DEGs were identified on Day 0 (L0V_vs_H0V) and Day 15 (L15V_vs_H15V) of Cd exposure, respectively, with 68 upregulated DEGs and 121 and 203 downregulated DEGs, respectively. There were nine common DEGs between the two groups. In the comparison groups between Day 0 and Day 15, the low-Cd-accumulating comparison group (L0V_vs_L15V) and the high-Cd-accumulating comparison group (H0V_vs_H15V) identified 249 and 321 DEGs, respectively, with 114 and 124 upregulated DEGs and 135 and 197 downregulated DEGs, respectively. There were 12 common DEGs between the two groups.

### 3.4. KEGG Enrichment Analysis of Differentially Expressed Genes

**Figure 2:**
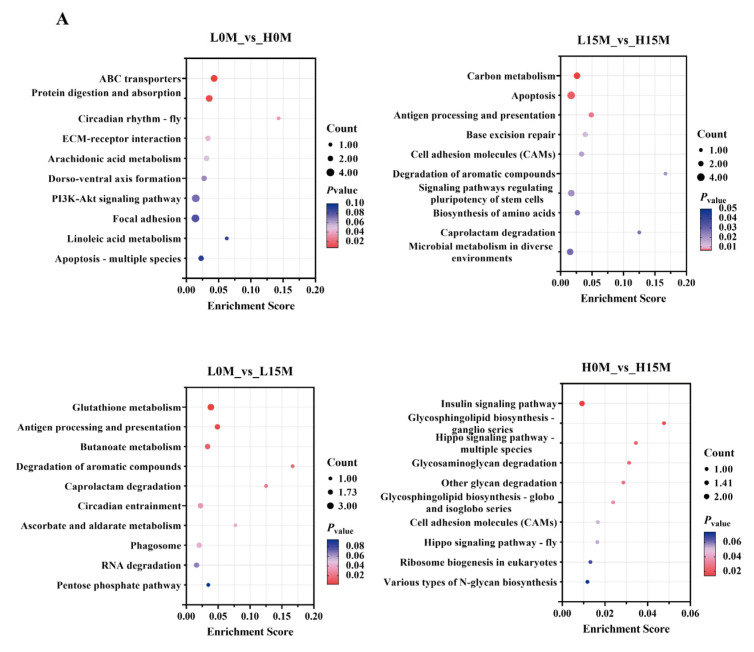

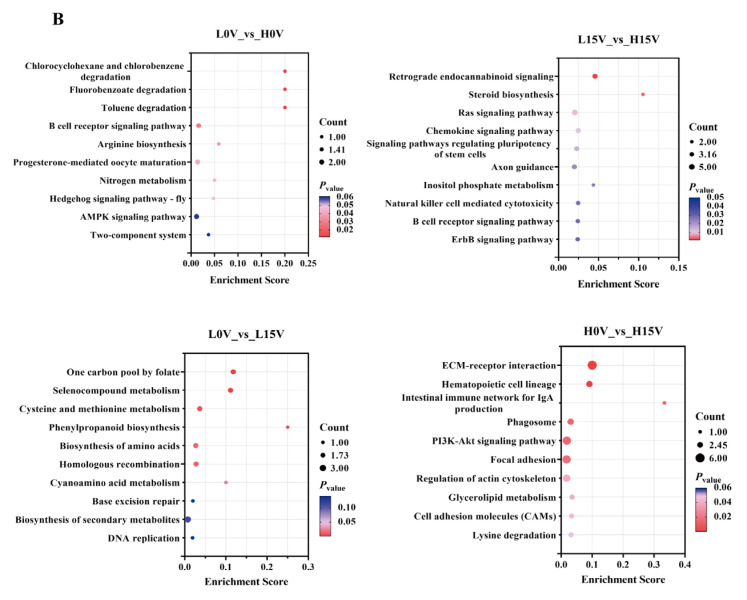
Bubble charts of KEGG enrichment for each comparison group under Cd exposure. (**A**) KEGG bubble plots of each comparison group in the mantle. (**B**) KEGG bubble plots of each comparison group in the visceral mass.

KEGG enrichment analysis of all DEGs revealed that 66 DEGs were significantly enriched in 62 metabolic pathways in the mantle and visceral mass of Portuguese oysters, with metabolic pathways (33.8%) and organismal systems (32.2%) accounting for the largest proportions (Figure 2). The top 10 enriched pathways included ABC transporters, ECM–receptor interaction, phagosomes, glycosaminoglycan degradation, chlorocyclohexane and chlorobenzene degradation, glutathione metabolism, selenocompound metabolism, biosynthesis of amino acids, antigen processing and presentation, and apoptosis (*p*-value < 0.05; Figure 2). Among these, ABC transporters, phagosomes, and glutathione metabolism were significantly associated with metal transport and detoxification.

### 3.5. Screening of Cd Accumulation-Related Genes and GWAS Joint Analysis

Based on KEGG enrichment analysis and Cd accumulation-related gene screening (Table 5), nine metal transport-related genes were identified, including *copt5.1*, *abca3*, *orct2*, *cac*, *harbi1*, *cacna1e*, *trpm3*, *lac24*, and *chrna10*. Except for *trpm3*, *chrna10*, and *harbi1*, the expression trends of these genes in different comparison groups were positively correlated with Cd accumulation levels, with significantly higher expression in HC compared to LC (*p*-value < 0.05). Furthermore, eight Cd detoxification-related genes were identified, including *sod3*, *cyp10*, *hspa12a*, *hspa12b*, *cyp4f22*, *pxdnl*, *chac1*, and *cyp20a1*. Among these, *sod3* was significantly downregulated after Cd exposure, with higher expression in LC compared to HC (*p*-value < 0.05). Three cytochrome P450 (CYP450) family genes showed significant differential expression only in the visceral mass. Transcriptome-GWAS joint analysis identified 13 genes with polymorphisms significantly associated with Cd content in Portuguese oysters, which also showed significant differential expression at the transcriptional level, including *edil3*, *orct2*, *zip1*, *harbi1*, *chac1*, *adar*, *spr*, *ankrd50*, *ccno*, *lac24*, *chrna6*, *hmcn1*, and *nachralpha2*.

### 3.6. qPCR Validation

**Figure 3:**
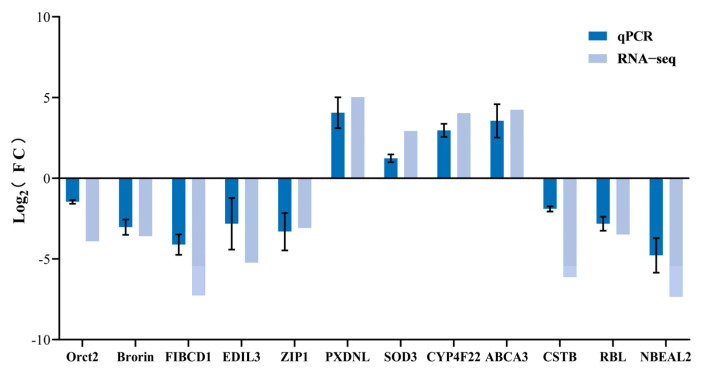
Verification of the relative expression levels of genes by *qPCR*. Fold change is the fold change of FPKM in RNA-seq or fold change of relative expression in *qPCR* (*p* < 0.05).

To validate the transcriptome data, 12 genes related to Cd accumulation and detoxification were selected for *qPCR* validation. The *qPCR* results (Figure 3) showed that the expression trends of the 12 DEGs were consistent with the transcriptome sequencing results, confirming the reliability of the data.

### 3.7. Alternative Splicing

**Figure 4:**
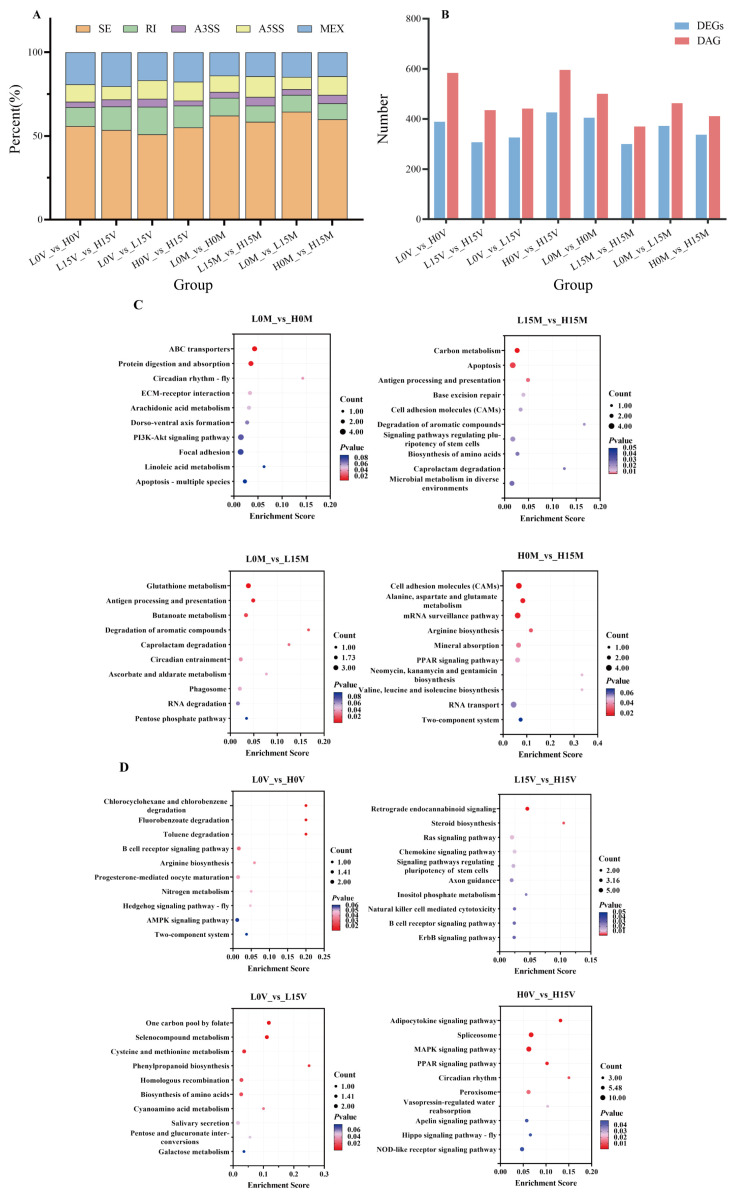
Alternative splicing (AS) events in each group. SE: skipped exon; MXE: mutually exclusive exons; A5SS: alternative 5′ splice site; A3SS: alternative 3′splice site; RI: retained intron. (**A**) Classification of AS in each comparison group. (**B**) Number of DEGs and AS events in each comparison group. (**C**) KEGG bubble charts of genes from differentially alternative splicing (DAS) events in the mantle. (**D**) KEGG bubble charts of genes from DAS events in the visceral mass.

A total of 77,615 alternative splicing (AS) events were identified in the mantle and visceral mass of Portuguese oysters. Using a false discovery rate (FDR) of less than 0.05 as the screening criterion, 3803 differentially alternative splicing (DAS) events were obtained. The DAS events were classified and summarized (Figure 4A), showing that among the five types of DAS events, the SE (skip exon) class dominated in both the mantle and visceral mass (50.9–60.3%), while the A3SS (alternative 3′ splice site) class consistently had the lowest number of DAS events. It is speculated that SE class AS events are widely present and play an important role in Portuguese oysters.

Further analysis of the differential results showed (Figure 4B) that in the mantle, the L0M_vs_H0M and L15M_vs_H15M groups obtained 501 and 370 DAS events, respectively, corresponding to 405 and 300 source genes. The L0M_vs_L15M and H0M_vs_H15M groups obtained 463 and 412 DAS events, respectively, corresponding to 373 and 337 source genes. In the visceral mass, the L0V_vs_H0V and L15V_vs_H15V groups obtained 584 and 435 DAS events from 389 and 307 genes, respectively. The L0V_vs_L15V and H0V_vs_H15V groups obtained 442 and 596 DAS events from 326 and 426 genes, respectively. KEGG enrichment analysis of all DAS source genes revealed 59 significantly enriched KEGG pathways (Figure 4C,D), including ABC transporters, carbon metabolism, glutathione metabolism, and MAPK signaling pathway. Using a screening criterion of *p*-value < 0.05 and |log2FC| > 1, 13 significantly expressed genes were identified, including glutamine synthetase 2 cytoplasmic (*gs2*), glucosamine-fructose-6-phosphate aminotransferase [isomerizing] 1 (*gfpt1*), and peroxisomal acyl-coenzyme a oxidase 1 (*acox1*). These 13 genes are suggested to play important roles in Cd accumulation and metabolism in Portuguese oysters.

## 4. Discussion

Bivalves universally exhibit tissue-specific enrichment of trace metals, irrespective of their metabolic essentiality [30]. In this study, Cd content in both the mantle and visceral mass of Portuguese oysters significantly increased following Cd exposure. Notably, the mantle demonstrated a higher Cd accumulation capacity than the visceral mass, as evidenced by its greater BAF, highlighting distinct tissue-specific accumulation patterns. Similar phenomena have been observed in other aquatic species. For instance, Cd distribution in palaemonid shrimp (*Palaemon macrodactylus*) follows the order [39]: gill > hepatopancreas > muscle, while in Chinese scallop (*Chlamys farreri*) [40], Cd levels are ranked as hepatopancreas > gill > mantle > adductor muscle. This tissue-specific partitioning likely reflects sequential processes of Cd absorption, transport, and storage in aquatic organisms.

Transcriptomics, a pivotal tool in functional genomics, enables the systematic dissection of cellular regulatory networks and the precise quantification of gene expression patterns under specific physiological conditions [41]. After Cd exposure, 8 Cd transport-related genes were identified in Portuguese oyster individuals with significantly different Cd accumulation capacities. Among these, *orct2* and *copt5.1* exhibited significant expression differences not only between high- and low-Cd-accumulating individuals but also displayed dynamic expression changes pre- and post-Cd-exposure. Furthermore, the differential expression of *sod3*, *cyp4f22*, *pxdnl*, *cyp10*, and *chac1* suggests potential roles in mitigating Cd exposure through the modulation of antioxidant defense systems and heavy metal detoxification pathways.

Cd has no known biological functions in organisms, so there are no specific transport channels for it. However, Cd can enter and exit cells through the transport systems of other essential metal elements (Cd^2+^, Ca^2+^, Zn^2+^) [42,43,44]. Multiple studies have demonstrated that Cd enrichment in plants and bivalves correlates with the activation of zinc–iron transporter proteins (ZIP family), which have been implicated in intracellular Cd transport [45,46,47]. For instance, Han et al. demonstrated that *zip2* overexpression elevates Cd levels in the leaves of *Sedum plumbizincicola* [48]. In bivalves, Wu et al. identified two ZIP family genes (*zip1* and *zip3*) significantly associated with Cd accumulation traits in Portuguese oysters through GWAS and Fst analyses [37]. Subsequent work by Li et al. showed that knockdown of *zip1* and *zip3* directly altered Cd content in oyster mantles (decrease and increase, respectively), leading to the hypothesis that *zip1* and *zip3* mediate Cd uptake and efflux in Portuguese oysters [46]. In the current study, *zip1* exhibited upregulated expression in high-Cd-accumulating individuals (L15M vs. H15M) following Cd exposure. This expression pattern aligns with Li’s functional prediction for *zip1*, further substantiating its role in promoting intracellular Cd transport through enhanced expression.

Based on prior GWAS and Fst screening results, we identified *orct2* as significantly correlated with Cd accumulation traits in Portuguese oysters. Existing research indicates structural homology between *orct2*, *oct1*, and *oct2*, all belonging to the major facilitator superfamily of membrane transport proteins [49,50,51]. While the functional characterization of *orct2* remains incomplete, *oct1* and *oct2* are well-established transporters of organic cations and pharmaceuticals. Notably, rat *oct1* mediates 1-methyl-4-phenylpyridinium (MPP^+^) transport [52], while human *oct2* internalizes Cd2+ with a Michaelis constant (Km) of 54 ± 5.8 μM [53]. In our study, *orct2* displayed significant differential expression between Portuguese oyster individuals with distinct Cd accumulation capacities (L0N vs. H0N) and pre- and post-Cd-exposure (L0N vs. L15N), with elevated expression strongly associated with high-Cd-accumulating phenotypes. We, therefore, propose that *orct2* participates in cellular Cd internalization, where its heightened expression enhances Cd absorption capacity in Portuguese oysters.

The copper transporter family (COPT) proteins have been established as high-affinity plasma membrane transporters mediating cellular copper uptake [54,55]. Zhang et al. conducted a transcriptomic study on rapeseed (*Brassica oleracea*) under Cd stress and found that COPT genes serve as critical pathways for Cd transport in cabbage seedlings [56]. Chen et al. further confirmed through the transcriptomic analysis of wheat under Cd stress that the expression of *copt3* promotes Cd absorption in wheat [57]. In this study, *copt5.1* showed significant differential expression between Portuguese oyster individuals with significantly different Cd accumulation capacities (L0W_vs_H0W) and pre- and post-Cd-exposure treatment (L0W_vs_L15W). Its high expression levels were highly correlated with the high-Cd-accumulation trait. Based on this, we speculate that *copt5.1* may provide more binding sites for Cd^2+^, thereby transporting Cd^2+^ into cells. Collectively, *zip1*, *orct2*, and *copt5.1* in Portuguese oysters may be involved in Cd absorption, and upregulated expression of these genes could lead to higher Cd accumulation in vivo.

Aberrant Cd accumulation in organisms poses serious threats to survival and development. Over long-term evolution, organisms have developed a series of adaptive mechanisms to mitigate Cd toxicity [28]. ATP-binding cassette (ABC) transporters represent the largest known superfamily of active transmembrane transporters, utilizing energy from ATP hydrolysis to drive metal ion transmembrane transport [58,59]. Multiple studies have demonstrated that ABC transporters enhance organismal tolerance to heavy metals, yet their mechanisms vary: *abcb1* (Cu) [60], ABCB25 (Al) [61], ABCC (GS-Pb) [62], and HMT-1 (PC-Ni) [63] sequester heavy metals or their complexes into vacuoles to improve tolerance; and in *Leishmania*, ABCG2 sequesters antimony–thiol conjugates into vesicles and expels them via exocytosis [64]. Similarly, ABC transporters act as Cd efflux pumps, facilitating cellular Cd extrusion [65,66]. In this study, *abca3* expression in the mantle was significantly higher in low-Cd-accumulating individuals compared to high-Cd-accumulating individuals (L0M_vs_H0M), consistent with functional inferences by Li [67] and Dong [30] that *abca3* upregulation promotes cellular Cd efflux, thereby reducing Cd accumulation in Portuguese oysters. Cytochrome P450 superfamily enzymes (CYPs) represent the most critical xenobiotic-metabolizing enzymes in organisms, oxidizing foreign substances via heme-bound iron ions to enhance water solubility and excretion [68]. Previous studies have linked CYPs to Cd metabolism: Cd stress induced increased CYP mRNA expression in piglets [68] (*cyp1a1*, *cyp7a1*, *cyp2b22*), thick shell mussel (*Mytilus coruscus*) [69] (*cyp3a*), and Manila clam (*Ruditapes philippinarum*) [70] (*cyp414a1*). In this study, *cyp4f22* was significantly upregulated in low-Cd-accumulating individuals (L0V_vs_H0V), suggesting its involvement in Cd extrusion pathways. Thus, high *cyp4f22* expression likely reduces Cd accumulation in Portuguese oysters.

Cd exposure causes severe oxidative damage to bivalve mollusks, and the antioxidant defense system plays a crucial role in scavenging ROS and resisting Cd toxicity [71]. Superoxide dismutase (SOD), a key component of the antioxidant defense system, can dismutate superoxide anion radicals (O_2_^−^) into oxygen (O_2_) and hydrogen peroxide (H_2_O_2_), thereby alleviating ROS damage induced by abnormal Cd accumulation [72]. Multiple studies have shown that upregulated *sod* expression enhances Cd tolerance in bivalves. For example, under Cd exposure, *sod* expression increased 28-fold in the Yesso scallop (*Mizuhopecten yessoensis*) [73] and was significantly elevated in *Ruditapes philippinarum* [74] and blue mussel (*Mytilus edulis*) [75]. Results revealed significant differential expression of *sod3* in Portuguese oyster individuals with distinct Cd accumulation capacities after Cd exposure (L15M_vs_H15M, L15V_vs_H15V). Prior to Cd exposure, no significant difference in *sod3* expression was observed between high- and low-Cd-accumulating individuals. However, after Cd exposure, *sod3* expression levels were significantly higher in low-Cd-accumulating individuals compared to high-Cd-accumulating individuals. These findings indicate significant differences in cellular detoxification strategies between oyster individuals with varying Cd accumulation capacities. The high expression of *sod3* in low-Cd-accumulating individuals may indicate more intense oxidative damage induced by Cd exposure, necessitating greater mobilization of SOD to mitigate such damage.

## 5. Conclusions

In this study, transcriptome sequencing technology was used to comparatively analyze the response mechanisms related to metal transport and detoxification in high- and low-Cd-accumulating individuals of Portuguese oysters, as well as before and after Cd exposure. DEGs were mainly enriched in pathways such as ABC transporters, phagosomes, glutathione metabolism, mineral absorption, MAPK signaling pathway, and hippo signaling pathway. We also identified genes related to heavy metal transport and detoxification, including *orct2*, *copt5.1*, *abca3*, *cyp4f22*, *sod3*, and *zip1*. Functional validation and research on these DEGs will help deepen the understanding of the transport and detoxification mechanisms of Cd in Portuguese oysters.

## Figures and Tables

**Table 1:** Classification patterns of comparison groups.

Time	Mantle (M)	Visceral Mass (V)
Day 0	L0M_vs_H0M	L0V_vs_H0V
Day 15	L15M_vs_H15M	L15V_vs_H15V
Day 0 to 15	L0M_vs_L15M	L0V_vs_L15V
	H0M_vs_H15M	H0V_vs_H15V
Low-Cd-accumulating comparison groups	L0M_vs_H0M L0M_vs_L15M	L0V_vs_H0V L0V_vs_L15V
High-Cd-accumulating comparison groups	H0M_vs_H15M L15M_vs_H15M	H0V_vs_H15V L15V_vs_H15V

**Table 2:** Primers used in this study.

Primer	Sequence (5′ to 3′)
*ef1α*-F	ACCACCCTGGTGAGATCAAG
*ef1α*-R	ACGACGATCGCATTTCTCTT
*orct2*-F	CCACAAACAGCTCCTATTTCGT
*orct2*-R	TCAGTGACATATGAGCGGTC
*zip1*-F	ATGCGCGTCATCTCCAACTT
*zip1*-R	AAAGAGGAGGAGGACGCTTG
*pxdnl*-F	TGAAGGTTCGAGGGTCAACG
*pxdnl*-R	TCGCGGAACTCCAGCATATC
*abca3*-F	AATGAGGTGGAGAGGCCTTG
*abca3*-R	CACTGGTTGGCCATCAAATCC
*edil3*-F	TGCTTGCTTTCCATGTGCATC
*edil3*-R	AACTGGACCTAGTCAGCGGT
*fibcd1*-F	CCGCGTAGGACTGATGGTTT
*fibcd1*-R	TGGAGCCCAGTTTCACACAA
*cstb*-F	CCAATAAGGTGTGGTGGGCT
*cstb*-R	TGTTCCAGCCACAATCTGAGT
*rbl*-F	CAATGTATGGGCGGGAGGAT
*rbl*-R	TCCAAATACAGAGTTCCGTGCT
*brorin*-F	CAGGTTGCCAGCAGTCATTA
*brorin*-R	CAGTGCGGCTTGTTAACTCA
*nbeal2*-F	CGATGCCTTGCGAGTTTGAAT
*nbeal2*-R	AGAATCCATTGCGGCGAGAA
*cyp4f22*-F	GAACGACCTTGGGAGATTGGA
*cyp4f22*-R	CACAGCTTCCGGCTGGTAT
*sod3*-F	GAAGCGACATACAGAGCGGA
*sod3*-R	AGTCTGACCCGGAATCTCCT

**Table 3:** The Cd content of mantle (M) and visceral mass (V).

Group	Value	M (μg/g)	V (μg/g)
Day 0	Max	7.97	21.87
	Min	2.92	5.71
	Mean ± SD.	4.68 ± 1.04	12.97 ± 3.5
Day 15	Max	58.84	43.92
	Min	8.65	13
	Mean ± SD.	21.02 ± 11.5	23.43 ± 7.03
	BAF	8.17	5.23

**Table 4:** The Cd content of low-Cd-accumulating individuals (LC) and high-Cd-accumulating individuals (HC).

Transcriptome Sample Grouping	Mean ± SD.	Transcriptome Comparison Group	Fold Change
L0V	7.13 ± 1.58	L0V_vs_H0V	2.98
H0V	21.22 ± 0.83	L15V_vs_H15V	2.54
L15V	13.49 ± 0.77	L0V_vs_L15V	1.89
H15V	34.29 ± 1.57	H0V_vs_H15V	1.62
L0M	3.48 ± 0.03	L0M_vs_H0M	2.10
H0M	7.32 ± 0.86	L15M_vs_H15M	5.05
L15M	9.54 ± 0.2	L0M_vs_L15M	2.74
H15M	48.16 ± 10	H0M_vs_H15M	6.48

**Table 5:** The list of potential key genes.

	Gene	Deception	Group
Cation (cadmium) transport	*copt5.1*	copper transporter 5.1-like isoform ×1	L0M_vs_H0M (down) L0M_vs_L15M (down)
	*abca3*	atp-binding cassette sub-family a member 3	L0M_vs_H0M (up)
	*harbi1*	putative nuclease harbi1 [*Crassostrea virginica*]	L15M_vs_H15M (up) L0M_vs_L15M (down) L0V_vs_L15V (down) GMAS
	*zip1*	zinc transporter zip1	L15M_vs_H15M (down) GMAS
	*orct2*	organic cation transporter-like protein	L0V_vs_H0V (down) L0V_vs_L15V (down) GMAS
	*trpm3*	transient receptor potential cation channel subfamily m member 3	L0V_vs_H0V (up)
	*cac*	voltage-dependent r-type calcium channel subunit alpha-1e	H0V_vs_H15V (down)
	*lac24*	laccase-24	L0M_vs_H0M (down) L0M_vs_L15M (down) GMAS
	*chrna10*	neuronal acetylcholine receptor subunit alpha-10	H0V_vs_H15V (up)
Detoxify	*hspa12b*	heat shock 70 kda protein 12a isoform x1	L0M_vs_H0M (down)
	*hspa12a*	heat shock 70 kda protein 12a-like	L0M_vs_H0M (down) L15M_vs_H15M (up) L0M_vs_L15M (up) H0V_vs_H15V (down)
	*sod3*	extracellular superoxide dismutase [cu-zn]	L15M_vs_H15M (up) L15V_vs_H15V (up)
	*pxdnl*	peroxidase-like protein	L0M_vs_L15M (up)
	*cyp4f22*	cytochrome p450 4f22	L0V_vs_H0V (up) L0V_vs_L15V (up)
	*cyp20a1*	cytochrome p450 20a1	L15V_vs_H15V (down)
	*cyp10*	cytochrome p450 10	H0V_vs_H15V (up)
	*chac1*	glutathione-specific gamma-glutamylcyclotransferase 1	L0M_vs_L15M (up) GMAS
Others	*spr*	sex peptide receptor	L0M_vs_H0M (up) H0M_vs_H15M (down) GMAS
	*adar*	double-stranded RNA-specific adenosine deaminase	L0M_vs_H0M (down) GMAS
	*ankrd50*	ankyrin repeat domain-containing protein 50	L0M_vs_H0M (down) GMAS
	*ccno*	cyclin-o	L0V_vs_L15V (up) GMAS
	*chrna6*	neuronal acetylcholine receptor subunit alpha-6	H0V_vs_H15V (up) GMAS
	*edil3*	egf-like repeat and discoidin i-like domain-containing protein 3	L0V_vs_H0V (down) L15V_vs_H15V (down) GMAS
	*hmcn1*	hemicentin-1	L0V_vs_L15V (down) GMAS
	*nachralpha2*	acetylcholine receptor subunit alpha-like	L0V_vs_L15V (up) GMAS

## Data Availability

Data will be made available on request.

## Supplementary Materials

The following supporting information can be downloaded at: https://www.mdpi.com/article/10.3390/ani15071041/s1.
